# Editorial: The Roles of Lipids in Immunometabolism: The Crosstalk Between Lipid Metabolisms and Inflammation

**DOI:** 10.3389/fcvm.2022.938535

**Published:** 2022-06-22

**Authors:** Jue Zhang, Wen Dai, Yiliang Chen

**Affiliations:** ^1^Versiti Blood Research Institute, Milwaukee, WI, United States; ^2^Department of Medicine, Medical College of Wisconsin, Milwaukee, WI, United States

**Keywords:** lipids, immunometabolism, atherosclerosis, chronic inflammation, innate immunity metabolic syndrome, cardiovascular disease

Mammalian cells contain a variety of lipid molecules and it was estimated that more than 1,000 lipid species can be found in one cell (1). Lipids constitute over 10% dry weight of a mammalian cell (2) and glycerophospholipids alone contribute to 20 ~ 25% of the dry weight in brain tissues (3). The heterogeneity of the cellular lipid molecules is in agreement with their diverse functions, ranging from supporting membrane structures (e.g., phospholipids, cholesterol, and glycolipids), energy storage (e.g., triglycerides), to intracellular and intercellular signaling (e.g., lipoprotein complexes, oxysterols, phosphoinositides, and prostaglandins). An emerging research field called immunometabolism has been inspired by the observations that abnormal cellular metabolism, including lipid metabolism, is often associated with an abnormal immune response (4). Alteration in lipid metabolism in immune cells (e.g., macrophages, dendritic cells, neutrophil, B cells, and T cells, etc) and non-immune cells (e.g., endothelial cells, smooth muscle cells, platelets) often play a significant role in systemic inflammation, leading to various diseases including atherosclerosis, diabetes, obesity, and cancer [Berkowitz et al.; Khafagy and Dash, (5, 6)]. Vice versa, pro-inflammatory cytokines and signaling regulate lipid metabolism. For example, TNF-α upregulated LDL receptor (LDLR) and downregulated scavenger receptor class B type-I (SR-BI), leading to cholesterol accumulation in human arterial endothelial cells (7). In another study, TNF-α attenuated ABCA1 expression through NF-κB pathway and reduced cholesterol efflux to HDL in human intestinal cells (8). Taken together, it is clear that lipid metabolism is closely coupled with inflammation.

Despite accumulating evidence indicating a crosstalk between lipid metabolism and inflammation, underlying molecular mechanisms under physiological and pathophysiological conditions remain poorly understood. Our contributors to this Research Topic have provided their novel data and ideas through both research and review articles, addressing the fundamental question how lipids cause health issues through the immune system.

## Lipoproteins in Regulation of the Immune System

As hydrophobic molecules, extracellular lipids often group together in extracellular vesicles or are associated with lipid-carrying proteins, forming lipoprotein complexes such as VLDL, LDL and HDL. In human circulation, LDL are the predominant lipoproteins, which are mostly cleared by LDLR in the liver *via* receptor-mediated endocytosis (9). Consistently, knocking out of LDLR in mice leads to very high plasma LDL levels and diet-induced atherosclerosis, which becomes one of the widely used atherosclerotic animal models (10). Proprotein convertase subtilisin/kexin type 9 (PCSK9) is an enzyme promoting degradation of LDLR, and therefore regulates plasma LDL cholesterol levels. Xia et al. summarized recent progress in understanding the regulation of PCSK9 expression and function and discussed how these mechanisms influence both lipoprotein metabolism and inflammation, as PCSK9 also degrades major histocompatibility protein class I in cancer cells. Similarly, Wu et al. provided an interesting review of recent findings that PCSK9 modulates inflammation through several pathways including TLR4/NF-κB signaling, lectin-like oxidized-LDL receptor-1 (LOX-1)-mediated pro-inflammatory responses, and induction of pro-inflammatory cytokines. Altogether, PCSK9 is a typical example that lipoprotein metabolism and inflammation process are coordinated and appears to be a promising target for therapeutic intervention of the atherosclerotic cardiovascular disease (CVD).

LDL are often modified due to oxidative stress in atherosclerosis conditions, leading to accumulation of oxidized LDL (oxLDL) and acetylated LDL (acLDL) within the vascular tissues (11). These modified LDL induce many pro-inflammatory responses in various immune cells. Liao et al. reported a novel mechanism that oxLDL inhibited a microRNA, miR-491-5P in THP-1-derived macrophages. MiR-491-5P silenced expression of matrix metalloproteinase 9 (MMP-9), which facilitated the pro-inflammatory responses such as reactive oxygen species (ROS) production, expression of pro-inflammatory cytokines TNF-α, IL-1β, and IL-6. As oxLDL reduced miR-491-5P levels, MMP-9 expression was elevated along with its downstream pro-inflammatory phenotypes (Liao et al.). In another study, oxLDL bound to a macrophage surface scavenger receptor CD36 and activated an intracellular protein kinase A (PKA), which phosphorylated vimentin, a type III intermediate filament protein (Kim et al.). PKA phosphorylated vimentin at Ser72, which directed intracellular CD36 trafficking to the plasma membrane and promoted CD36-mediated oxLDL uptake as well as foam cell formation. Consistently, knocking out vimentin resulted in 57% less atherosclerotic lesion formation in *Apoe* null mice on high fat diet for 15 weeks (Kim et al.). While the pro-atherogenic role of CD36 in macrophages has been widely documented (6, 12), Rekhi et al. showed that CD36 in endothelial cells (EC) also contribute to atherosclerosis by mediating fatty acid uptake, leading to dysfunctional endothelium. The group has generated EC-specific CD36 knockout mice and crossed them with LDLR knockout mice (EC CD36^−/−^/LDLR^−/−^). They found that female but not male EC CD36^−/−^/LDLR^−/−^ mice were protected from diet-induced atherosclerosis, suggesting a sex-dependent atherogenic effect in EC CD36 (Rekhi et al.). Besides CD36, circadian genes Bmal1 expression appeared to limit oxLDL uptake and maintain EC functions in a hindlimb ischemia mouse model. Bmal1 inhibited inflammation by activating anti-inflammatory cytokine IL-10 expression and promotes angiogenesis through VEGF signaling (Xu et al.).

AcLDL is another major form of modified LDL that facilitate macrophage intracellular cholesterol accumulation and foam cell formation. Willemsen et al. have found that acLDL loading in macrophages specifically suppressed type-I interferon (IFN) signaling and IFN-β secretion. This phenotype was also observed in monocytes isolated from familial hypercholesterolemia patients by RNA sequencing analysis. Thus, this study has provided a potential connection between cellular cholesterol metabolism and inflammatory signaling in innate immune cells (Willemsen et al.). In another study, Zhao et al. revealed a crosstalk between glycolysis pathway and lipid metabolism that affected macrophage phenotype during atherogenesis. They showed that solute carrier family 37 member 2 (SLC37A2), a protein regulating glycolysis is required for alternative activation of macrophages to mediate anti-inflammatory responses. Hematopoietic cell-specific deletion of SLC37A2 in LDLR knockout mice lead to increased plasma lipid during atherogenesis as well as more atherosclerosis plaque development (Zhao et al.).

Compared to LDL and their derivatives that generally impose detrimental effects, HDL are considered beneficial to the human health and the most studied cardioprotective function of HDL is their ability to promote cholesterol efflux from peripheral cells (13). In agreement with this notion, HDL-mediated cholesterol efflux capacity (CEC) or reverse cholesterol transport (RCT) in macrophages is impaired in metabolic diseases such as atherosclerosis (14) and non-alcoholic fatty liver disease (15), both of which are associated with chronic inflammation. Moreover, impairment of CEC has been reported in autoimmune and pro-inflammatory conditions including acute phase reaction (16), rheumatoid arthritis, and systemic lupus erythematosus (17), indicating the involvement of HDL functions in regulation of the immune system. Recent evidence further indicate that systemic inflammation and autoimmune disease conditions reciprocally impact the composition and functions of HDL particles (18) as well as HDL/apoA-I plasma levels (19). Therefore, it is more and more clear that HDL is one of the major players in our immune system and more mechanistic studies are urgently needed in this field.

Al-Jarallah and Babiker reported a novel anti-hypertensive and a cardioprotective effect of HDL in spontaneously hypertensive rats after myocardial ischemia/reperfusion. Mechanistically, the effect was dependent on cardiac SR-BI, a known HDL receptor. Chronic HDL treatment protected cardiac myocytes by reducing autophagy and inflammation. Autophagy is critical for lipid metabolism in both immune and non-immune cells during atherosclerosis (20) and it is a process coupling extracellular stress signals, cellular lipid handling and sensing, and immune cell activation (21). Therefore, the study by Al-Jarallah and Babiker on chronic HDL effects on autophagy deserves further exploration. However, while the beneficial effects of HDL on cardiovascular system are widely recognized, many questions regarding the underlying molecular mechanisms remain to be answered. For example, do HDL directly counteract LDL effects on vascular cells or immune cells through shared receptors and/or downstream effectors? Alternatively, since HDL reduce peripheral cell cholesterol levels by mediating cholesterol efflux or reverse cholesterol transport, do HDL impose beneficial effects on immune system indirectly through alleviating the cellular lipid burden? In addition, similar to LDL, HDL can be chemically modified, which appear to impair their physiological functions (22, 23). It would be highly interesting to further explore the impact of the modified HDL *in vivo* and how they alter systemic inflammation in metabolic diseases.

## Lipid Metabolism and DYS-Regulated Inflammation in Human Diseases

The lipid-laden macrophages in the atherosclerotic plaques are good examples of the connection between a defective lipid metabolism and abnormal inflammatory responses. Those macrophages show ectopic intracellular neutral lipid accumulation, accompanied by elevated secretion of pro-inflammatory cytokines such as IL-1β, TNF-α, MCP-1, and IL-6 (24). Thanks to the recent advancement in single cell RNA sequencing technologies combined with proteomics methods, the lipid-laden macrophages are observed in other human diseases such as cancer (25, 26), obesity (27), and non-alcoholic fatty liver disease (NAFLD) (28). *Via* genetic manipulation combined with pharmacological intervention that reduce lipid-laden macrophages, people show that those cells are actively involved in systemic inflammation during the disease development. These studies further emphasize the contributing role of abnormal lipid metabolism, especially in immune cells, in dys-regulated inflammation.

While the underlying molecular mechanisms have been widely characterized (29), Lee-Rueckert et al. summarized and broadened the view of lipid-laden macrophages beyond atherosclerosis. They discussed how the phenotypic and functional plasticity of macrophages become entangled in both atherosclerosis and cancer development. In fact, contrary to the conventional view that those macrophages simply facilitate progression in atherosclerosis and cancer, macrophage accumulation of lipid may be a response toward anti-inflammatory phenotypic switch through transcriptional reprogramming. If so, it may stimulate novel ideas targeting lipid-laden macrophages in either disease (Lee-Rueckert et al.).

Many recent studies have provided direct evidence that lipid species result in dys-regulated immune system in human. Patients with non-alcoholic fatty liver disease (NAFLD) are at increased risk of developing atherosclerosis and related CVD. Hoebinger et al. summarized and focused on the role of oxidized lipids that act as danger signals to drive pro-inflammatory processes and disease progression. Similarly, Karnati et al. used quantitative lipidomic analysis and demonstrated that altered lipid species (e.g., lysophosphatidylcholine) were associated with pro-inflammatory cytokines in the serum of human patients with Takotsubo Syndrome, an acute cardiac syndrome with increased inflammation (Karnati et al.). It should be noted here that human individual variation in many serum lipid species is high and may require a large number of samples in order to detect difference among groups. The power of this study is relatively low (262 individuals from three groups) and the data acquired here may be interpreted with caution. However, as Karnati et al. managed to show difference in lysophosphatidylchline, application of quantitative lipidomics appears to be a promising tool for a comprehensive study of serum lipid profiles. In another study, Berkowitz et al. reported that ceramide, one class of sphingolipid, plays a causative role in both type 2 diabetes and CVD. Finally, Khafagy and Dash reviewed the current knowledge of etiology and pathogenesis of inflammation in obesity-associated CVD. Animal and human data both indicate that adipose tissue, a specialized lipid storage tissue, is involved in hyperlipidemia and systemic inflammation in obesity. Based on human genetic and pharmacological studies, while anti-inflammatory treatment reduces CVD, off-target effects such as increased infection limit its broad therapeutic application, which warrants future studies on mechanistic link between lipid metabolism and systemic inflammation. It is our belief that this knowledge is critical for designing novel drugs targeting lipid metabolic enzymes because, in many disease settings, abnormal lipid metabolism may be the real driving force of inflammation.

## Author Contributions

YC determined the structure and drafted the editorial. JZ and WD edited and approved the final version. All authors contributed to the article and approved the submitted version.

## Funding

This work is supported by MCW New Faculty Startup Fund and NIH grants R01HL153397 (to YC).

## Conflict of Interest

The authors declare that the research was conducted in the absence of any commercial or financial relationships that could be construed as a potential conflict of interest.

## Publisher's Note

All claims expressed in this article are solely those of the authors and do not necessarily represent those of their affiliated organizations, or those of the publisher, the editors and the reviewers. Any product that may be evaluated in this article, or claim that may be made by its manufacturer, is not guaranteed or endorsed by the publisher.
